# Wearable Cardioverter Defibrillator as a Treatment in Patients with Heart Failure of Various Aetiologies—A Series of Ten Cases Within the BIA-VEST Registry

**DOI:** 10.3390/jcm13247686

**Published:** 2024-12-17

**Authors:** Małgorzata Kazberuk, Piotr Pogorzelski, Łukasz Kuźma, Anna Kurasz, Magdalena Róg-Makal, Urszula Matys, Justyna Tokarewicz, Paweł Kralisz, Sławomir Dobrzycki

**Affiliations:** Department of Invasive Cardiology, Medical University of Białystok, 15-089 Białystok, Poland

**Keywords:** wearable cardioverter defibrillator, sudden cardiac death, cardiovascular disease

## Abstract

**Background/Objectives:** Sudden cardiac death (SCD) remains a major global health concern and represents one of the most common causes of mortality due to cardiovascular diseases. The wearable cardioverter–defibrillator (WCD) is an innovative, non-invasive medical device designed to provide continuous heart monitoring and immediate defibrillation in patients at risk for SCD. The study aimed to assess the efficacy of WCD usage in patients awaiting decision on therapy with implantable cardioverter–defibrillators (ICDs). **Methods:** We explored the clinical applications, benefits, and limitations of WCD usage within the BIA-VEST registry in Poland over the years 2021–2023. The study included 10 patients with a mean age of 49.1 ± 12.02 years. **Results:** All patients demonstrated good tolerance and compliance with the LifeVest WCD, wearing it for an average of 93.1 days, about 22.8 h per day (95.7% of the time). No interventions from LifeVests were recorded, and there were no effective, ineffective, or inadequate discharges. After the first follow-up echocardiography, five patients still required ICDs. Due to improved LVEF and overall condition in six out of ten patients undergoing WCD bridge therapy, ICD implantation was finally waived. **Conclusions:** The WCD acts as a bridge to therapy, such as ICD implantation or cardiac surgery, and may be particularly beneficial for patients with transient or evolving conditions, such as structural heart diseases and life-threatening ventricular arrhythmias.

## 1. Introduction

Sudden cardiac death (SCD) is defined as natural death due to cardiac causes, manifested by a sudden loss of consciousness and death up to one hour after the onset of symptoms. Timing and the sudden and unexpected onset of death play a decisive role here, while the heart disease may or may not be previously known [1]. It is difficult to assess the incidence of SCD, but by gathering data from the United States, it can be established that SCD, including out-of-hospital cardiac arrests (OHCAs), can claim between 185,000 and 450,000 lives per year (7–18%) [2]. Chugh et al. stated that SCD is behind 4 to 5 million deaths per year worldwide [3]. In only 50% of SCD events is heart disease diagnosed before the incident [4,5]. Although SCD prevention is a high-priority health issue, in the European Union, there is very little reliable data on the number of incidents of SCD, as well as every OHCA [2]. According to Heart Disease and Stroke Statistics—2022 Update: A Report From the American Heart Association, the 2022 survival of OHCA to hospital discharge turned out to be only 9% with a post-hospital discharge survival rate of 7% with good functioning of the patient [6].

### Risk Stratification of SCD

The clinical assessment of patients should accompany daily management with the aim of prevention of SCD. Among comorbidities, renal insufficiency has been proven to significantly contribute to a patient’s risk of SCD [7]. The CADILLAC Risk Score allows the assignment of patients to low, intermediate, and high 1-year mortality risk categories founded on clinical assessment of renal insufficiency, Kilip class II/III, final TMI flow 0–2, age > 65 years, anaemia, and three-vessel disease with baseline LVEF < 40% identified as the most powerful predictor of mortality. A patient with reduced LVEF and at least one of the listed factors is immediately classified as high risk. Additionally, post-percutaneous coronary intervention (PCI) patients are considered to be at high risk of SCD. In the high-risk group, the most fatal are the first 3 months after angioplasty [7].

Coronary artery disease (CAD) and heart failure (HF) are the leading causes of SCDs, followed by cardiomyopathies and inherited arrhythmias [8]. In order to determine heart-specific risk factors, echocardiographic evaluation with the assessment of left ventricle ejection fraction (LVEF), electrocardiogram (ECG), and magnetic resonance imaging (MRI) are needed [9]. Additionally, SCD risk factors include a number of components associated with cardiovascular diseases according to the Framingham Risk Score and Heart Score [10]. The likelihood of ischemic heart disease in the general population can be stratified based on such scales, yet currently, there are no SCD-specific risk panels [11]. According to Empana et al., not only does the risk of SCD increase over age, but also the incidence is higher among men as compared with women [2]. In the population after the age of 35, the risk of SCD increases significantly [12]. However, according to 2017 American Heart Association (AHA) guidelines, there is no linear correlation between age and SCD, and the risk of SCD may decrease after 75 years of age [11]. The incidence of SCD is particularly high during the first months after acute myocardial infarction (MI), especially among patients with low LVEF. In cases of MI, high-risk complex ventricular arrhythmias, newly diagnosed severe HF, or patients awaiting heart transplantation, preventing SCD is crucial. However, immediate qualification for implantable cardioverter–defibrillator (ICD) implantation is not recommended [13]. Possible options for securing patients until an ICD implantation include anti-arrhythmic therapy, advanced telemetry, or wearable cardioverter–defibrillators (WCDs). Amiodarone is a leading drug suitable for patients with contraindications to device therapy that stabilises electrical activity and prevents arrhythmias, although it was reported to have limited effectiveness in preventing SCD and potential for serious side effects, such as thyroid toxicity or lung fibrosis [14]. Some companies are developing new technologies of advanced telemetry. These are still emerging alternatives and might not yet be widely available [15,16]. However, the use of a WCD might be a method of choice for SCD prevention.

WCDs are becoming increasingly common and widely available, as they are said to be successful in the prevention of SCD soon after MI [17]. ICDs in patients at high risk of cardiac arrest are an essential method of prevention months to years after MI [18]. Therefore, WCDs are becoming a bridge between the incidence of MI and the decision to implant an ICD [19].

We are presenting a series of ten cases involving high-risk patients treated with the use of WCDs in the prevention of SCD. The goal of this study was to determine the efficacy of WCDs during the period before ICD implantation.

The current literature reveals substantial knowledge gaps concerning WCDs. Addressing these areas could enhance the understanding and optimisation of WCD use. Our study aims to provide clarity on optimal patient selection for WCD therapy beyond broad risk categories (e.g., post-MI, recent cardiomyopathy diagnosis) and determine which subgroups of patients benefit most (e.g., based on EF, arrhythmia type).

## 2. Materials and Methods

Our single-centre retrospective, observational case study focused on indications and usage of WCDs as well as follow-up therapeutic measures in patients due to HF after MI, myocarditis, as well as in patients with dilated cardiomyopathy. The aim of this study was to demonstrate the effectiveness of using WCDs as a bridging therapy at a critical time for prevention of SCA and to identify clinical case examples of the use of WCDs.

Ten patients admitted to the LifeVest program were treated in the Department of Invasive Cardiology, Medical University of Białystok, Poland, in the years 2021–2023. All of them separately received a professionally guided session on WCD usage by qualified staff of the LifeVest (ZOLL Lifecor Corp., Pittsburgh, PA, USA) with an attending physician present. All the patients understood the purpose of its usage and gave their informed consent to use the WCD.

Our department is one of the largest centres in Poland that provides WCD treatment. Currently, the registry includes 10 patients, each with a minimum of one year of follow-up, as detailed in the following section. Patient data confidentiality was ensured by restricting access to the study database to authorised personnel only. Patient data were securely stored and handled, ensuring the highest standards of confidentiality throughout the study.

### 2.1. Selection of the Patients

Every patient who qualified for the study met the minimum age of 18 years and was hospitalised in the Department of Invasive Cardiology at the University Teaching Hospital, Medical University of Białystok, Poland. ECG, echocardiography, chest X-ray, laboratory tests, and coronary angiography were performed in all patients. Comorbidities, patients’ medical history, and other relevant characteristics were taken into account when qualifying for WCD.

### 2.2. Recommendations for WCD Therapy

Guideline-directed medical therapy (GDMT) was implemented in all patients, appropriately tailored to their clinical status and comorbid conditions. The indications for WCD were considered for patients who presented the following conditions:

- patients after acute MI with temporarily elevated risk of life-threatening arrhythmias waiting for the decision on ICD implantation;
- patients with myocarditis or dilated cardiomyopathy with severe impairment of ventricular systolic function associated with high risk of ventricular tachycardia (VT)/ventricular fibrillation (VF);
- patients initially qualified for an ICD who have contraindications for implantation;
- patients waiting for cardiac transplantation, using WCD as a bridge therapy.

## 3. Results

### 3.1. Patients’ Medical History

Patient #1—A 52-year-old patient, without history of chronic diseases, was referred to the cath lab with a diagnosis of anterior wall STEMI complicated by prehospital sudden cardiac arrest (SCA) in the VF mechanism after successful resuscitation. Simultaneous PCI of the left anterior descending artery (LAD) and implantation of a drug-eluting stent (DES) was performed. In a follow-up echocardiography performed after several days, there was a slight improvement in contractility from LVEF = 24% to LVEF = 31%.

A WCD was prescribed as a bridge to further therapy due to the patient’s condition and increased risk of SCD, with follow-up information provided in Table 1. Patient #2—The patient, a young, 35-year-old male with unsuccessfully treated bronchitis, was referred to the hospital for a chest X-ray, which showed inflammatory changes in the right lung subclavian field with enlarged cardiac cavities. In view of positive troponin in laboratory results, the patient was transferred to the Invasive Cardiology Department.

On admission, the patient was haemodynamically stable, reporting resting dyspnea, with heart rate (HR) 120/min and blood pressure (BP) 140/100 mmHg. ECG showed sinus tachycardia 120/min and features of left ventricle (LV) hypertrophy. Bedside echocardiography demonstrated significant enlargement of the left heart cavities, with LVEF = 13–15% with thrombotic material in the lumen of the LV.

Due to the patient’s condition and elevated risk of SCD, a WCD was used as a temporary measure, with further clinical follow-up provided in Table 1.

Patient #3—A 59-year-old patient with no medical history, who had been involved in competitive sports for many years, was admitted directly to the catheterisation laboratory with an anterolateral wall STEMI at 14 h from the onset of chest pain. The ECG showed atrial fibrillation with a QRS complex rate of 135/min, ST-segment elevation, and deep Q-waves over the anterior wall.

When echocardiography revealed left ventricular wall hypertrophy with severely impaired LVEF of 18%, dobutamine was added to the treatment. ECG and echocardiography details are provided in Table 2.

Coronary angiography showed LAD filled with thrombus, with subsequent redistribution of thrombus to Cx during thrombectomy. PCI of LAD with DES implantation was performed. Due to persistent continuous chest pain aggravated by breathing, separation of pericardial plaques in echocardiography and increases in C-reactive protein (CRP), colchicine, and antibiotic were added to the treatment. In a week’s time, stage II treatment, PCI of obtuse marginal artery (OM) with DES implantation was performed. The ECG recorded the evolution of an anterolateral MI with persistent ST elevation of the anterior wall. In the control ECHO, a slight improvement in LVEF of 22% was observed.

Given the patient’s condition and the increased risk of SCD, a WCD was used as a bridge to further therapy. Further clinical follow-up details are shown in Table 1.

Patient #4—A 49-year-old patient was admitted due to pulmonary oedema.

On admission, the patient was sweaty, dyspneic, and haemodynamically stable, with BP 106/79 mmHg. ECG showed a sinus rhythm of 126/min, pathologic left axis deviation, and persistent ST elevation in V1–V4. Echocardiography revealed severely impaired LVEF of 18%. For details of ECG and echocardiography, see Table 2.

Coronary angiography showed significant stenosis of the LAD, left circumflex artery

(LCx), and right coronary artery (RCA). The patient was consulted by the Heart Team and qualified for re-evaluation. Depending on the improvement in LVEF—coronary artery bypass grafting (CABG) or heart transplantation would have been considered.

Due to the high risk of SCD in the mechanism of ventricular arrhythmias, the patient was protected with a LifeVest defibrillation vest during the period until improvement was observed in LVEF. For further clinical follow-up details, see Table 1.

Patient #5—A 54-year-old male patient with no chronic treatment to date was transferred to the catheterisation laboratory with a diagnosis of anterior wall STEMI for primary PCI. For the past week, the patient had suffered from recurrent chest pains, aggravated for approximately four days, with the most severe about 2 h before admission. The patient was a smoker for 45 years (20/day) with no family history of cardiovascular conditions or comorbidities—see Table 2.

The patient presented significantly impaired exercise tolerance and NYHA III with LVEF = 18% on discharge. In view of the patient’s condition and the increased risk of SCD, the patient was qualified for a LifeVest defibrillation vest as a bridge therapy to ICD implantation.

Patient #6—A 35-year-old female patient, previously without a history of chronic disease, was transferred from the Cardiology Department for treatment of cardiogenic shock in the course of STEMI of the inferolateral wall. Echocardiography revealed LVEF = 12%. Coronary angiography performed at the referring centre showed LCx with thrombus and critical LAD lesions with thrombus—most likely due to spontaneous dissection (SCAD). PCI of LCx and LAD with implantation of 2 DES was performed. After the procedure, VF occurred three times—twice interrupted by defibrillation and once interrupted by metoprolol bolus. The patient qualified for subcutaneous cardioverter defibrillator (S-ICD) implantation and was secured with a LifeVest until the procedure.

Patient #7—A 64-year-old patient with suspected ACS, history of HF, persistent atrial fibrillation (AF), and diabetes mellitus (DM) type 2 was admitted to the Unit due to an episode of loss of consciousness, preceded by a feeling of dyspnoea and significant weakness. The ECG performed by the paramedics showed VT 243/min. Haemodynamic stabilisation was achieved with effective electrocardioversion.

On admission, the patient was haemodynamically stable, without stenocardia or resting dyspnoea, BP 119/80 mmHg. ECG recorded sinus rhythm of 77/min, pathologic left axis deviation, left anterior hemiblock (LAH), and signs of non-ST-elevation MI (NSTEMI). Systolic murmur was present over the whole heart. Auscultation revealed bilateral crackles at the base of the lungs. Echocardiography showed significant aortic stenosis with AVA 0.9 cm^2^ and LVEF = 45%. Coronary angiography showed generalised non-significant atherosclerotic lesions.

Before admission, the patient had developed diabetic foot due to poorly controlled DM type 2, which resulted in the necessity of amputation of the first toe of the left foot with the metatarsophalangeal joint.

The patient was temporarily disqualified from ICD implantation because of non-healing of the amputated toe. Therefore, the Heart Team decided to secure the patient with a WCD until the indication for ICD implantation. Further clinical follow-up details are shown in Table 1.

Patient #8—A 48-year-old female patient with post-inflammatory cardiomyopathy and recurrent VT (arrhythmia rate 110–175/min, periodically unstable requiring cardioversion) was admitted to the Department due to suspected infection of the newly-implanted ICD locus. Previously, the patient had undergone ICD implantation and removal of the system due to infection of the ICD locus on the left side.

Echocardiography revealed severely impaired LV systolic function with LVEF 32%. In laboratory tests, there were no features of generalised inflammation. The ICD system was removed, revealing purulent content in the device locus. During hospitalisation, the patient developed haemodynamically unstable VT of 175/min requiring electrical cardioversion.

Considering recurrent haemodynamically unstable VT in the patient without an implanted ICD, the patient was secured with a LifeVest.

Patient #9—A 31-year-old patient with a history of chest pain persisting approximately for 4 days, most severe the night before admission, with a diagnosis of STEMI of the anterior wall of the heart in evolution, was transferred by the emergency medical team for diagnosis and invasive treatment. On admission, the patient was haemodynamically stable with BP 129/86 mmHg without preserved chest pain.

ECG revealed sinus rhythm of 88/min, intermediate axis, ST elevation over the anterior wall with QS present in V1–V3, and negative T in I, aVL. Bedside cardiac echo showed extensive contractile abnormalities from LAD vascularisation and thrombus in the LV apex. Coronary angiography with intravascular ultrasound (IVUS) showed proximal LAD stenosis with massive thrombus and vessel dissection and TIMI-2 flow. In the CICU, the patient reported pericardial pain on control echo imaging, suggestive of early post-infarction pericarditis, with LVEF = 26%, and left ventricular thrombus 32 × 32 mm. Intensified treatment of heart failure was administered, colchicine, dual antiplatelet therapy with ticagrelor, low-molecular-weight heparin in therapeutic dose, and a 24-hour infusion of abciximab was administered after termination of cangrelor therapy. A follow-up coronary angiography showed a significant reduction of the thrombus in the LAD, without significant narrowing of the vessel lumen. On follow-up echocardiography, the thrombus was reduced, with no improvement in left ventricular systolic function.

The patient was secured with the LifeVest system until the next hospitalisation and the decision of ICD implantation.

Patient #10—A 63-year-old patient with a diagnosis of NSTEMI, multivessel coronary artery disease, heart failure with reduced ejection fraction (LVEF = 15%), and newly diagnosed paroxysmal AF (without anticoagulant treatment) was transferred to the Department in order to perform percutaneous treatment using mechanical left ventricular support with the Impella system. In the past, the patient had NSTEMI, which was treated conservatively due to lack of the patient’s consent for CABG. On admission, the patient was in good general condition, haemodynamically stable with BP 141/98 mmHg. ECG showed sinus rhythm 59/min, pathologic left axis deviation, LAH. The admission physical examination revealed signs of stasis in the pulmonary circulation and lower limb oedema. In Cathlab, a complex effective IVUS-controlled procedure with Impella support of PCI of LAD, left main coronary artery (LM), and LCx was carried out with rotablation and implantation of two DESs. The patient was further qualified for conservative treatment of the CAD. During follow-up, the patient remained stable and angina complaints did not recur. HF pharmacotherapy was optimised. A decision on eligibility for ICD implantation for primary prevention of SCD will be made after clinical and echocardiographic follow-up after scheduled readmission. Due to the high risk of life-threatening arrhythmia, the patient was secured with the Zoll LifeVest system until qualification for permanent ICD.

Details of ECG and echocardiography of all patients are shown in Table 2.

All patients were fitted with a Zoll LifeVest defibrillation vest.

### 3.2. Primary Results

The mean age of the study population was 49.1 ± 12.02. The report on patients’ data and follow-up after the period of WCD use is shown in Table 1.

In our ten-case series of patients treated with LifeVests, we noted good tolerance of the devices. Every patient showed good compliance and a very high mean wear time—an average of 93.1 ± 17.4 days, for 22.8 h/day (95.7% of the time).

There were no LifeVest interventions recorded in the BIA-VEST registry patients. There were no incidents of either effective or ineffective discharges nor incidents of inadequate discharge as well. In one patient, a non-sustained ventricular tachycardia (nsVT) was recorded, yet there was no discharge since the patient’s VT was self-limiting. Thanks to real-time monitoring, one patient was diagnosed with AF on ECG during follow-up. Also, patient #2 was able to self-record the event of bradycardia of 40/min during morning hours.

### 3.3. Follow-Up of the BIA-VEST Patients

Due to improvement in LVEF and lack of indications, ICD implantation was finally waived in 6 out of 10 patients undergoing bridge therapy.

After careful analysis of the overall clinical presentation of patient #7, the LifeVest protection period was extended after the balloon aortic valvuloplasty procedure. Taking into account the increase in LVEF and the absence of complaints even with greater exertion, it was decided to postpone ICD implantation in patient #10. Patients #4 and #6 were able to safely await full surgical revascularisation while being protected from SCD. ICDs were finally implanted in four patients (#2, #3, #8 and #9)

## 4. Discussion

We reported a series of ten cases of WCD usage due to various indications. There are still not enough studies on the efficacy of the WCD. The increasing use of the WCD is described on the basis of cohort studies [20,21]. WCDs have been proven to be effective in detecting ventricular arrhythmias (VT and VF) and providing the necessary therapy in the form of electrical discharge [22]. In addition to the relatively small number of patients who receive this type of therapy, further difficulty in observing the efficacy of WCD appears to be due to the low percentage of patients with discharge while wearing it [21].

Currently, several important cohort studies have been published demonstrating the effectiveness of WCDs, including studies from Germany and the USA [23,24], as well as The Wearable Defibrillator Investigative Trial (WEARIT) and the Bridge to ICD in Patients at Risk of Sudden Arrhythmic Death (BIROAD) studies, which were the first to demonstrate the clinical relevance of protecting patients with such a device.

The VEST trial [25] showed significant reduction in total mortality in the group that received the WCD compared with the control group (3.1% vs. 4.9%; *p* = 0.04). However, a reduction in cardiac deaths or deaths from ventricular arrhythmias was not found to be statistically significant. Yet, of the 48 patients who died in the VEST study, as many as 36 were not wearing the WCD at the time of the incident.

Also, the WEARIT/BIROAD study may be an example on which further development and refinement of WCD technology could be based to anticipate and eliminate potential problems with incorrect application of electrodes to the patient’s body and other factors modifying the correct functioning of the appliance [26].

The study by Chung et. al. demonstrated the efficacy of WCDs, with discharges effectively stopping ventricular arrhythmias (a single discharge interrupted 99% of arrhythmic attacks) and up to 90% survival after VT/VF [24]. The WEARIT-II study showed 5 effective discharges/100 patient-years, and the German registry study reported 8.4 discharges/100 patient-years [27,28]. The high rate of sVT/VF at 3-month follow-up in high-risk patients without indications for implantation of a cardioverter–defibrillator suggests that the WCD can be used for temporary protection against SCA. The study demonstrated a high first-year survival rate among patients protected by LifeVests (96% of all patients and 90% in the group with a malignant arrhythmia). The study provided evidence on the efficacy of WCD use in cases of risk of early heart failure complications after a cardiovascular incident [28].

Since 1998, when the first paper on the use of WCDs was published [29], valuable findings from cohort studies have been accumulated.

Survivors of SCA are at high risk of recurrent VT or VF, with recurrence rates increasing each year since the event [30]. Formerly, antiarrhythmic drug (AAD) therapy was the method of choice for secondary SCD prevention combined with Holter monitoring or programmed ventricular stimulation [31]. The CASCADE study evaluated amiodarone vs. other AADs for secondary prevention in 228 survivors of out-of-hospital cardiac arrest. Patients receiving amiodarone showed improved survival free of cardiac death or resuscitated VF at 2 years (82% vs. 69%) and sustained benefits after 6 years [32]. Amiodarone was demonstrated to lower the risk of SCD, making it a suitable treatment option for patients ineligible for or without access to ICD therapy for SCD prevention [14]. However, amiodarone therapy was proven to carry a higher risk of thyroid toxicity and pulmonary fibrosis [14,33].

Current guidelines advise against implanting an ICD within the first 3 months following revascularisation, as LVEF improvement remains uncertain during this period [34]. Recommendation IIb states that WCDs can be an applied therapy in patients in the early phase after MI in selected clinical situations [25]. In addition, for patients awaiting heart transplantation, WCD may be considered as additional protection during the waiting period for surgery [23]. Current guidelines still recommend implanting an ICD at least 40 days after MI and 90 days after CABG [35]. Numerous randomised trials have proven early ICD implantation after MI not to be beneficial, as it did not reduce mortality in patients soon after MI [13,36]. Studies on vests show that it is worthwhile to protect patients with dual therapy, i.e. pharmacological and WCD, while waiting for the decision to implant an ICD [34].

In comparison to the ICD, the WCD has the potential to reduce the delivery of inadequate therapy by the patients themselves. Although there is a risk of inappropriate shock, the WCD device uses an algorithm to determine which rhythm to defibrillate [37]. Each patient is educated in the management of wearing the WCD, including how to defer discharge when the device misinterprets the rhythm, as in our patient during a well-tolerated VT. The patient prevented electrical cardioversion, while the VT resolved spontaneously without further consequences. This is made possible by a standardised time of less than 1 min until discharge when the patient is able to prevent discharge on their own. This significantly reduces the number of inadequate therapy deliveries by the device. As a result of the device’s external electrodes, interference with the signal collected by the electrodes is often observed [37,38]. Despite the existence of the WCD’s therapy-withholding response buttons, inappropriate therapy remains the most common associated complication [26]. Unfortunately, the device can interpret many artefacts, noise, supraventricular tachycardia, or short-term VT in ECG records as a rhythm to intervene. The VEST study noted a 0.6% occurrence of inadequate discharges [25]. The German study [27] of 6043 patients revealed a remarkably low index of inappropriate shocks observed in 26 patients (0.4%), an incidence rate of 2.3 (95% CI, 1.5–3.4) per 100 patient-years, nor have any patient deaths been reported in the course of device discharge.

The therapy itself was well tolerated by patients from this study, which was proven by very good compliance—the mean time of WCD wearing per day was 22.5 h, while the study by Garcia et. Al. showed that younger age was associated with lower compliance. Such low compliance may be associated with a lifestyle that is incompatible with WCD usage. Education is probably a key factor that can improve adherence [21].

Moreover, Olgin et al. found that married status, white ethnicity, prior PCI, and history of cardiac arrest were linked with better compliance [20]. A study by Schuhmann et al. demonstrated worsening of the quality of life while wearing the WCD. Patients were reported to have sleep disturbances and limitations in daily activities [39]. Despite the discomfort that WCDs bring on a daily basis, the authors of many studies continue to confirm the need to consider the use of WCDs, as the current guidelines also indicate [40,41]. As it appears, the length of WCD use is dependent on the cause of the SCD risk as well as further decisions on treatment. The analysis by Wäßnig et al. included a distinction of the number of days of WCD use according to the cause of risk of sudden cardiac death [27]. In our follow-up, we noticed an increase in LVEF similar to other studies [13,42]. However, the physical burden of wearing the device 24/7 can negatively impact quality of life (QoL). Patients often report discomfort, inconvenience in daily routine activities or self-care [39]. Additionally, the constant reminder of their cardiac condition may exacerbate stress for some individuals, making the experience emotionally taxing. In the study by Weiss et. al., symptoms of depression and anxiety were prevalent in patients with WCDs and were strongly associated with lower health-related QoL [43]. Nevertheless, some parallel studies did not find WCD use as a cause of negative patient-reported health outcomes [44]. The continuing advancement of telemedicine is becoming increasingly relevant in many areas of medicine, including cardiology and WCD therapy. The development of accurate and continuous monitoring of WCD patients by telemedicine may increase the sense of security and QoL, indirectly, in affected patients [45].

The widespread use of WCDs has significant policy implications for healthcare delivery, regulation, reimbursement, and patient access. Addressing these issues could optimise WCD use and improve patient outcomes. Variability in reimbursement policies may impact access and equity, especially in low-resource settings. Policies should advocate for universal insurance coverage for high SCD-risk patients, regardless of socioeconomic status or location. Cost-effectiveness benchmarks and analyses should guide reimbursement, regulatory approval, and large-scale procurement. The costs of WCDs, along with the question of their cost-effectiveness in terms of quality-adjusted life years and the varying cost-effectiveness thresholds in different countries, may create barriers to WCD access [46]. There are reports on the cost-effectiveness of WCD bridge therapy in specific populations, such as patients after ICD explantation or awaiting ICD reimplantation [47,48]. Botto et. al. suggests that the use of WCDs in post-MI patients is both clinically beneficial and cost-effective. WCDs may help optimise resource utilisation, possibly in any healthcare system [49]. WCD use may also be associated with reduced costs in many healthcare areas, including inpatient stays, physician services, and skilled nursing facility utilisation [50].

Competitive pricing and manufacturer negotiations could reduce costs for public health systems. Standardised patient education protocols are crucial for safe use, while psychological support services, hotlines, and support networks can enhance adherence and address challenges in WCD use.

## 5. Conclusions

WCD therapy may benefit patients with conditions like MI, myocarditis, or dilated cardiomyopathy, by protecting against SCD in specific scenarios of temporary or reversible risk. Post-MI patients with reduced LVEF (<35%) who are at high SCD risk within 90 days but not eligible for ICDs, as well as those with newly diagnosed cardiomyopathy awaiting LVEF improvement, are key groups. Patients with severe infections or device-related complications temporarily unable to undergo invasive procedures may also benefit. WCD offers a non-invasive interim solution to mitigate SCD risk while awaiting risk resolution or therapy optimisation. Large registries and multi-centre studies are needed to validate these proposals.

## 6. Future Directions

Future research and studies on WCDs should address undetermined factors in order to clarify the indications, the length of WCD therapy, patients’ adherence, as well as therapy effectiveness and long-term benefits. Conduction of large-scale prospective cohort studies is needed to evaluate WCD outcomes in different populations (e.g., ischemic vs. non-ischemic cardiomyopathy, post-MI patients with moderate LVEF reduction). Use of biomarkers (e.g., NT-proBNP, troponins) and advanced imaging (e.g., cardiac MRI) could enable SCD risk stratification and prediction of WCD benefits. It is also necessary to explore WCD use in underinvestigated populations such as women and elderly patients, who are often underrepresented in clinical trials. Performing randomised controlled trials (RCTs) is relevant to compare outcomes with different durations of WCD use (e.g., 30, 60, or 90 days) in specific populations of patients at risk of SCD.

It is also necessary to conduct trials testing enhanced algorithms for arrhythmia detection to reduce false-positive and false-negative arrhythmia detections and inappropriate shocks, in order to aspire towards more accurate detection. Presenting patients with the possibility of support from mobile health platforms could improve patient engagement and adherence. Investigation towards linking WCDs with wearable sensors (e.g., for activity, blood pressure, or oxygen saturation) may provide a more comprehensive picture of patient health. Last, but not least, conducting multi-centre registries and observational studies to capture real-world WCD utilisation and challenges across different healthcare systems is needed to assess feasibility, barriers, and outcomes. These focused studies could fill critical gaps in WCD research and help refine guidelines for its use in preventing SCD.

## 7. Study Limitations

The major limitation of the study is the small number of patients with different indications for WCD therapy, making it difficult to generalise the effectiveness of WCD therapy. At the same time, the detailed description of these patients and their clinical data provide valuable insights into the diverse characteristics and profiles of individuals who may benefit from WCD protection. This diversity allows for a broader understanding of how WCDs can be applied in varying clinical scenarios, potentially guiding more personalised and effective use of these devices in future patient populations.

## Figures and Tables

**Table 1:** Characteristics of patients and course of the disease treated with bridge therapy with LifeVest.

Patient	Sex/Age	Indications	LVEF on Admission	NYHA on LifeVest Application	LVEF on LifeVest Application	Coronary Angiography/PCI	AS/IAS	Control EF	Time of Usage (Days)	NYHA After WCD Therapy	LVEF After WCD Therapy	Implantation of ICD and Follow-Up
#1	M/52	STEMI	24%	III	31%	PCI RCA, LAD Obstructed initial segment of LAD with thrombus and 95% stenosis of RCA	None	42%	76 days	II	N/A	No ICD implantation
#2	M/35	Myocarditis	13%	III	15–22%	Not indicated	None	25%	94 days	I	30%	Implantation of ICD
#3	M/59	STEMI	16%	II	18–22%	PCI LAD, PDA LAD filled from the outlet with thrombus	None	22%	68 days	II	28%	Implantation of ICD; 2 years after implantation—exacerbation of HF with EF = 18%, NT-pro-BNP = 5350 pg/mL, presence of SVT supraventricular arrhythmias, 9% recording and approximately 7000 supraventricular extrasystoles
#4	M/49	dilated cardiomyopathy, pulmonary oedema	18%	III	18–22%	Significant stenosis of the LAD, 1 and 2 Dx, LCx and RCA/OM	None	N/A	96 days	I	33%	No ICD implantation; CABG: LIMA-DG-LAD, SV-RPD-RPL
#5	M/54	STEMI	14%	III	18%	PCI LAD	None	33%	88 days	I	42%	No ICD implantation
#6	F/35	STEMI with SCAD Haemodynamically unstable VT	12%	II	20%	PCI LAD, LCx	None	22%	72 days	II	22%	No ICD implantation; CABG: LIMA-LAD, SV-OM, SV-RCA
#7	M/64	Haemodynamically unstable VT	45%	III	48%	Coronary arteries without significant atherosclerotic lesions	None	55%	97 days	II	48%	No ICD implantation; BAV. Prolongation of the LifeVest therapy
#8	F/47	Myocarditis	27%	II	25–32%	Not indicated	None	33%	107 days	II	35%	Implantation of ICD PET scan to exclude possible sites of infection
#9	M/31	STEMI	26%	III	26%	PCI with IVUS, stenosis of the proximal LAD with massive thrombus and dissection of the vessel and TIMI-2 flow	None	22%	114 days	I	32%	Implantation of ICD
#10	M/63	NSTEMI	15%	II	15%	PCI with IVUS of LAD/LM/Cx with rotablation and implantation of 2 DES	None	15%	119 days	I	34%	No ICD implantation

Abbreviations: AS, appropriate shock; BAV, balloon aortic valve valvuloplasty; CABG, coronary artery bypass grafting; Dx, diagonal branch; EF, ejection fraction; IAS, inappropriate shock; ICD, implantable cardioverter–defibrillator; IVUS, intravascular ultrasound; LAD, left anterior descending artery; LIMA, left internal mammary artery; LCx, left circumflex artery; PCI, percutaneous coronary intervention; PDA, posterior descending artery; RCA, right coronary artery; SCAD, spontaneous coronary artery dissection; STEMI, ST-elevation myocardial infarction; SV, saphenous vein bypass; OM, obtuse marginal; RPD, right posterior descending artery; RPL, right posterolateral branch; VT, ventricular tachycardia; WCD, wearable cardioverter–defibrillator.

**Table 2:** Imaging tests and comorbidities of patients treated with bridge therapy with Life-Vest.

Patient	ECG on Admission	ECHO on Admission	Chest X-Ray on Admission	Comorbididies
#1	Sinus rhythm of about 100/min, pathologic right axis deviation, RBBB, nsVT (4 beats visible), single extra ventricular beats	EF = 24% Akinesis of the apex, apical segments of the lateral wall and IVS. Akinesis of the apical and middle segment of the anterior wall. Hypokinesis of the basal segment of the anterior wall. Hypokinesis of the basal segment of the anterior wall and basal and medial segments of the lateral wall. Expansion of the left heart cavities. No features of significant valvular defect. Moderately impaired LV diastolic function. Severely impaired LV systolic function.	Bedside X-ray. Congestive thickening in the pulmonary fields. Thickened congestive hilum. Medial shadow dilated.	HFrEF, hypercholesterolemia, atherosclerosis, no family burden of cardiac diseases, BMI 28 kg/m^2^
#2	Sinus tachycardia of 124/min, intermediate axis, left ventricular hypertrophy, ST segment depression with negative T-waves in V5–V6	EF = 13%. A 35 × 12 mm balloting thrombus attached to the interventricular septum and inferior wall. Thrombus in the apex and apical segment of the anterior wall measuring 20 × 12 mm. Generalised LV wall hypokinesis. Heart fully dilated. Dilated ascending aorta. No evidence of significant valvular defect. Severely impaired LV systolic function.	Fluid in the left diaphragmatic-final angle. Congested pulmonary hili. Adjacent thickening in the subclavian field of the right lung—inflammatory changes. Lungs with increased pattern. Silhouette of the heart enlarged.	Hypercholesterolaemia, cigarette smoker, no family burden of cardiac diseases, BMI 31.1 kg/m^2^
#3	Atrial fibrillation with QRS complex frequency 110/min, pathologic left axis deviation, LAH, ST-segment elevation in I, aVL, V2–V6, Q-wave in V1–V6, ST-segment reductions in II, III, aVF, negative T-wave in V2–V5	EF = 22% Akinesis of the apex, apical and middle segments of the interventricular septum and anterior wall, apical segments of LV walls. Hypokinesis of the basal segment of the IVS and anterior wall. Hypokinesis of the lateral wall. Expanded left heart cavities. LV wall hypertrophy. No features of significant valvular defect. Moderately impaired LV diastolic function. Severely impaired LV systolic function.	Bedside X-ray. Diaphragm on the right side indistinct, on the left side visualised with cardiac silhouette. Right lung field with reduced pneumatisation with presence of atelectatic and inflammatory changes. Right hilum accentuated. Silhouette of the heart enlarged.	HFrEF, pericarditis, paroxysmal AF, primary hypertension, mixed hyperlipidaemia, chronic kidney disease G2, mitral and tricuspid regurgitation, family history of atherosclerosis, BMI 31.2 kg/m^2^
#4	Sinus rhythm of 126/min, pathologic left axis deviation, QS in V1–V3, no R-wave progression in V4–V5, persistent ST-segment elevation in V1–V4	EF = 18% Akinesis of the IVS, apical segments of the LV walls. Akinesis of the inferior wall and middle segment of the anterior wall. Widened LA and LV cavity. LV wall hypertrophy. No features of significant valvular defect. Moderately impaired LV diastolic function. Severely impaired LV systolic function.	Pulmonary fields with markedly enhanced lining, with the presence of fine nodular shadows at the base of the right lung. Features of redistribution of the upper lobe vessels on the left side. Right hilum dilated, vascularly thickened. Silhouette of the heart enlarged.	HFrEF, mixed hyperlipidaemia, insulin-dependent DM with cardiovascular complications, smoker, no family burden of cardiac diseases, BMI 25.95 kg/m^2^
#5	Sinus tachycardia of 116/min, persistent ST elevation in V1–V6, aVL (up to about 5mm in V2–V4), II, III, aVF (max. 8 mm in V2–V3) with features of necrosis—QS bands in V2–V6, LAH	EF = 18% Within the left ventricular apical aneurysm—thrombus 19 × 14 mm, dyskinesis of the apical and middle segments of the LV, hyperkinesis of the basal segments of the LV walls, LV cavity dilatation, LV hypertrophy, severely impaired LV systolic function, pericardial fluid in the pericardial sac without threatening tamponade.	Chest X-ray taken in sitting position, on partial inspiration. Diaphragm and angles free. Lung fields without fresh focal changes.	HFrEF, primary hypertension, atherosclerosis, mixed hyperlipidaemia, cigarette smoker, no family burden of cardiac diseases, BMI 29.7 kg/m^2^
#6	Sinus tachycardia of 145/min, low QRS complex voltages in limb leads, persistent ST. elevation in V1–V3, single VEBs	EF = 12% Akinesis of the middle and apical segments of the lateral and the anterior wall, of the apical and middle segments of the inferolateral wall, of the middle segment of the inferior wall, of the middle segment of the interventricular septum, hypokinesis of the apical segments of the other walls Conclusions- Widened LV cavity. Mild mitral and tricuspid regurgitation. Severely impaired LV systolic function. Small amount of fluid in the left pleural cavity.	Bedside X-ray, left diaphragm with obliterated outline, small amount of fluid in left diaphragmatic angle. Lung congestion. Silhouette of the heart not dilated.	HFrEF, hypercholesterolemia, mitral regurgitation, no family burden of cardiac diseases, BMI 23.4 kg/m^2^
#7	Sinus rhythm of 77/min, pathologic left axis deviation, LAH, biphasic T in V3–V4, negative T in II, III, aVF, V5–V6	EF = 45% General hypokinesis. Dilated left heart cavity and RA. Mild LV wall hypertrophy. Features of significant aortic stenosis—AVA 0.9 cm^2^. Moderately impaired LV wall relaxation. Mildly impaired LV systolic function.	Bedside X-ray taken in sitting position, Left lung field with diminished transparency. Right lung field without fresh focal or infiltrative changes. Aortic arch with presence of atherosclerotic plaques.	HFmrEF, paroxysmal AF, severe aortic stenosis, poorly controlled DM with vascular complications, smoker, father suffered from MI, BMI 28.4 kg/m^2^
#8	Effective atrial pacing 70/min (AAI pacemaker) intermediate axis, negative T wave in I, II, aVL, V5, V6, single extra ventricular exitation and a pair of variably shaped ventricular excitations	EF = 32% Globally impaired relaxation. Visible pacemaker leads in right heart cavities without obvious features of infectious endocarditis. No features of infectious endocarditis on valves. Pericardium without fluid. General LV wall hypokinesis. Cardiac dimensions within normal limits. No features of significant valvular defect. Impaired LV systolic function. Mildly impaired LV diastolic function.	Lung fields with marked lining pattern. Single scar band in the lower right lung field. Medial shadow within normal limits. In the projection of the middle field of the right lung, a shadow of the pacemaker is visible—the ends of the electrodes in the RA and RV projections.	HFrEF, AF, Hashimoto disease, liver insufficiency, inflammatory lesions of the oral cavity with caries and gingival reaction, no family burden of cardiac diseases, BMI 31.6 kg/m^2^
#9	Sinus rhythm of 88/min, intermediate axis, ST elevation over the anterior wall with QS present in V1–V3, negative T in I, aVL	EF = 26% Extensive contractile abnormalities from LAD vascularization. Thrombus in the LV apex. Severely impaired global systolic function. Dilated LV with regional wall motion abnormalities - akinesis of the apical segments of the LV walls and the apex, akinesis of the middle segment of the anterior wall and interventricular septum, hypokinesis of the remaining segments of the LV walls. Small mitral valve regurgitation	Bedside X-ray taken in sitting position. Right diaphragmatic dome free, left with partially obliterated outline. Decreased pneumatization in the lower field of the left lung. Vascular recesses thickened congestively. Dilated heart silhouette.	HFrEF, mixed hyperlipidaemia, alcohol dependence syndrome, smoking 20 cigarettes per day, BMI 33.9 kg/m^2^
#10	Sinus rhythm of 59/min, pathologic left axis deviation, LAH, negative T in I, aVL, V4–V6, non-specific intraventricular conduction disturbances, features of LV wall hypertrophy	EF = 15% IVC with limited respiratory mobility. Presence of fluid in the left pleural cavity. Hypokinesis of the basal and middle segment of the inferior, lower-lateral wall and IVS. Hypokinesis of the remaining LV wall segments. Heart fully dilated, without features of a significant valvular defect. Severely impaired LV systolic and diastolic function.	Bedside X-ray, taken in sitting position, asymmetrical. Lung fields with increased lining with features of congestive lesions. Heart silhouette enlarged.	HFrEF, hypertention, hypercholesterolaemia, past NSTEMI, paroxysmal AF, BMI 26.2 kg/m^2^

Abbreviations: AAI, atrial single chamber pacing; AF, atrial fibrillation; BMI, body mass index; DM, diabetes mellitus; ECG, electrocardiography; EF, ejection fraction; HFrEF, heart failure with reduced ejection fraction; IVC, inferior vena cava; IVS, intraventricular septum; LAH, left anterior hemiblock; LV, left ventricle; nsVT, non-sustained ventricular tachycardia; RA, right atrium; RBBB, right bundle branch block; RV, right ventricle; VEB, ventricular extrasystolic beat.

## Data Availability

Anonymized data might be shared upon reasonable request.
